# Increased Microerythrocyte Count in Homozygous α^+^-Thalassaemia Contributes to Protection against Severe Malarial Anaemia

**DOI:** 10.1371/journal.pmed.0050056

**Published:** 2008-03-18

**Authors:** Freya J. I Fowkes, Stephen J Allen, Angela Allen, Michael P Alpers, David J Weatherall, Karen P Day

**Affiliations:** 1 Peter Medawar Building for Pathogen Research and Department of Zoology, University of Oxford, Oxford, United Kingdom; 2 Department of Medical Parasitology, New York University School of Medicine, New York, New York, United States of America; 3 The Weatherall Institute of Molecular Medicine, University of Oxford, John Radcliffe Hospital, Oxford, United Kingdom; 4 PNG Institute of Medical Research, Goroka, Papua New Guinea; Imperial College London, United Kingdom

## Abstract

**Background:**

The heritable haemoglobinopathy α^+^-thalassaemia is caused by the reduced synthesis of α-globin chains that form part of normal adult haemoglobin (Hb). Individuals homozygous for α^+^-thalassaemia have microcytosis and an increased erythrocyte count. α^+^-Thalassaemia homozygosity confers considerable protection against severe malaria, including severe malarial anaemia (SMA) (Hb concentration < 50 g/l), but does not influence parasite count. We tested the hypothesis that the erythrocyte indices associated with α^+^-thalassaemia homozygosity provide a haematological benefit during acute malaria.

**Methods and Findings:**

Data from children living on the north coast of Papua New Guinea who had participated in a case-control study of the protection afforded by α^+^-thalassaemia against severe malaria were reanalysed to assess the genotype-specific reduction in erythrocyte count and Hb levels associated with acute malarial disease. We observed a reduction in median erythrocyte count of ∼1.5 × 10^12^/l in all children with acute *falciparum* malaria relative to values in community children (*p* < 0.001). We developed a simple mathematical model of the linear relationship between Hb concentration and erythrocyte count. This model predicted that children homozygous for α^+^-thalassaemia lose less Hb than children of normal genotype for a reduction in erythrocyte count of >1.1 × 10^12^/l as a result of the reduced mean cell Hb in homozygous α^+^-thalassaemia. In addition, children homozygous for α^+^-thalassaemia require a 10% greater reduction in erythrocyte count than children of normal genotype (*p* = 0.02) for Hb concentration to fall to 50 g/l, the cutoff for SMA. We estimated that the haematological profile in children homozygous for α^+^-thalassaemia reduces the risk of SMA during acute malaria compared to children of normal genotype (relative risk 0.52; 95% confidence interval [CI] 0.24–1.12, *p* = 0.09).

**Conclusions:**

The increased erythrocyte count and microcytosis in children homozygous for α^+^-thalassaemia may contribute substantially to their protection against SMA. A lower concentration of Hb per erythrocyte and a larger population of erythrocytes may be a biologically advantageous strategy against the significant reduction in erythrocyte count that occurs during acute infection with the malaria parasite Plasmodium falciparum. This haematological profile may reduce the risk of anaemia by other *Plasmodium* species, as well as other causes of anaemia. Other host polymorphisms that induce an increased erythrocyte count and microcytosis may confer a similar advantage.

## Abstract 2

Karen Day and colleagues show that increased microcytic erythrocyte count may contribute substantially to the protection of α^+^-thalassaemia-homozygous children against severe malaria anaemia.

## Editors' Summary

**Background.:**

Mutations (changes in the DNA that encodes proteins) continually arise within human populations. Harmful mutations that affect an individual's ability to reproduce usually disappear, but most other mutations persist at a low frequency. Some mutations, however, protect their human carriers against specific disease-causing organisms, and consequently occur at high frequencies in human populations that live in places where these organisms are common. For example, the inherited blood disorder α^+^-thalassemia, which is common in Africa and Southeast Asia, provides protection against malaria, a parasitic disease that occurs in tropical and subtropical parts of the world. α^+^-Thalassemia is caused by the loss of one or more of the genes that encode the α chains of hemoglobin, the red blood cell (erythrocyte) protein that carries oxygen around the body. These α chains are normally encoded by four genes, two on each Chromosome 16 (all chromosomes come in pairs). People with heterozygous α^+^-thalassemia lack one copy of the α chain gene and have a –α/αα genotype (genetic makeup). People with homozygous α^+^-thalassemia lack one copy of the gene on each chromosome (they have a –α/–α genotype) and have mild “microcytic anemia,” a condition characterized by increased numbers of abnormally small erythrocytes (microcytosis) that contain reduced amounts of hemoglobin.

**Why Was This Study Done?:**

Paradoxically, although homozygous α^+^-thalassemia causes mild anemia, it provides protection against severe malarial anemia, a potentially fatal complication of malaria. Malaria parasites cause anemia because they multiply inside erythrocytes and rupture them. Scientists originally thought that α^+^-thalassemia protects against malaria by interfering with the parasite's ability to infect erythrocytes, but the evidence collected so far does not support this hypothesis. In this study, therefore, the researchers have investigated whether the microcytosis and increased erythrocyte count associated with α^+^-thalassemia might be responsible for the protection that this blood disorder provides against severe malarial anemia. Specifically, they asked whether this hematological (blood) profile protects against severe malarial anemia because people with the –α/–α genotype lose less hemoglobin for a given degree of malaria-induced erythrocyte loss than do those with the normal genotype.

**What Did the Researchers Do and Find?:**

A study done in the mid 1990s in children living on the north coast of Papua New Guinea (where 68% of the population has α^+^-thalassemia) showed that homozygous α^+^-thalassemia protects against severe malaria. To investigate why, the researchers re-analyzed the genotype-specific reduction in erythrocyte counts and hemoglobin levels associated with acute malarial disease in these children and developed a simple mathematical model to predict hemoglobin levels after malaria infection. They found that when malarial infection reduced the number of erythrocytes per liter of blood by more than 1.1 × 10^12^ (the average measured loss of erythrocytes in this population because of malaria was 1.5 × 10^12^ per liter), children with homozygous α^+^-thalassemia lost less hemoglobin than did those with the normal genotype. Furthermore, children with homozygous α^+^-thalassemia needed a 10% greater reduction in their red blood cell count than children with the normal genotype for their hemoglobin levels to fall below the value that defines severe malarial anemia.

**What Do These Findings Mean?:**

These findings suggest that the increased number of abnormally small erythrocytes associated with homozygous α^+^-thalassemia might be responsible for the protection against severe malarial anemia that this blood disorder provides, because more erythrocytes have to be destroyed by the parasite to reduce hemoglobin concentrations to a dangerous level than in people with the normal genotype. In other words, a lower concentration of hemoglobin per erythrocyte coupled with a larger population of erythrocytes might be advantageous in the face of the large reduction in erythrocyte numbers caused by infection with malaria parasites. The researchers note that their study population was infected with only one type of malaria parasite (Plasmodium falciparum), but speculate that the hematological profile associated with α^+^-thalassemia might also prevent other *Plasmodium* species causing anemia. Futhermore, they suggest, other mutations that increase the erythrocyte count and cause microcytosis might protect against severe malaria anemia in a similar fashion.

**Additional Information.:**

Please access these Web sites via the online version of this summary at http://dx.doi.org/10.1371/journal.pmed.0050056.

- The MedlinePlus encyclopedia contains pages on thalassemia and on malaria (in English and Spanish)
- Detailed information is available on thalassemia (including useful links to other resources) from the US National Heart Lung and Blood Institute, from the US National Human Genome Research Institute, from the Cooley's Anemia Foundation, and from MedlinePlus
- The US Centers for Disease Control and Prevention provide information on malaria (in English and Spanish)
- Information is also available from the World Health Organization on malaria (in English, Spanish, French, Russian, Arabic, and Chinese)

## Introduction

The heritable haemoglobinopathy α-thalassaemia is one of the most common monogenic disorders of humans [1]. The different forms of α^+^-thalassaemia result from deletions (3.7 or 4.2 kb) or point mutations in one of the duplicated α-globin genes (αα/αα) on Chromosome 16 [1]. The heterozygous (−α/αα) and homozygous (−α/−α) states for α^+^-thalassaemia are characterised by lower haemoglobin (Hb) concentration, mean cell volume (MCV) and mean cell Hb (MCH), but increased erythrocyte count compared with normal individuals [1–3]. This mild hypochromic, microcytic anaemia is more pronounced in individuals homozygous for α^+^-thalassaemia compared to heterozygous individuals [1–3].

Haldane's proposal that the high frequencies of thalassaemias in malaria endemic regions were due to natural selection by malaria [4] is consistent with the strong geographical correlation between the frequency of α^+^-thalassaemia and malaria in the Pacific region [5]. It has been suggested that α^+^-thalassaemia protects by a direct interaction between the parasite (Plasmodium falciparum) and the altered thalassaemic erythrocyte, resulting in reduced parasite load [6]. However, in vitro experiments have failed to consistently demonstrate either a reduced ability of the malaria parasite to grow in and/or invade thalassaemic erythrocytes [7–17], and epidemiological studies have failed to demonstrate any consistent effect of α^+^-thalassaemia against P. falciparum density [18–22]. These findings argue against an altered physical interaction between the erythrocyte and the parasite.

Case-control studies in Papua New Guinea (PNG) [18] and Africa [19,20,22,23] have demonstrated the protective effect of α^+^-thalassaemia against severe malaria. Most studies show that α^+^-thalassaemia homozygotes have considerable protection against SMA compared to heterozygotes [18,20,22,23], although one study showed equal odds of heterozygotes and homozygotes developing SMA [19]. We hypothesised that this striking protection against SMA may be due to the microcytosis and increased erythrocyte count in children homozygous for α^+^-thalassaemia.

We have reported previously a case-control study of children living in the north coastal region of PNG that showed that the odds ratio for SMA was 0.34 (95% confidence interval [CI] 0.16–0.73) in homozygous α^+^-thalassaemia compared to normal individuals [15]. In the present study, we explore the consequences of a range of reductions of erythrocyte count on genotype-specific Hb levels by modelling the observed haematological data from the original case-control study.

## Materials and Methods

### Study Site

The north coast of PNG provides a unique site to investigate the protective effect of α^+^-thalassaemia against malaria. The frequency of α^+^-thalassaemia is very high (68%) [1], and other host erythrocyte polymorphisms such as Southeast Asian ovalocytosis and glucose-6-phosphate dehydrogenase deficiency are relatively uncommon in this population (<7%) [18,21]. Importantly, sickle cell trait, a haemoglobinopathy proposed to be a serious confounder in epidemiological studies of α^+^-thalassaemia in Africa [24,25], is absent in PNG.

### Data Collection

In the case-control study, children with acute malaria were recruited from outpatient clinics and Madang General Hospital between October 1993 and February 1996 [18]. In children admitted to hospital, malaria was defined as a febrile illness with any degree of P. falciparum parasitaemia (as they had often received antimalarial treatment before admission), but without an alternative cause of illness identified during detailed clinical and laboratory investigation. Some of these children developed one or more severe manifestations of malaria, defined according to World Health Organization criteria [26,27]. For each case of severe malaria, a control child living in the community was selected randomly and individually matched to the index case for age, sex, ethnicity, season, and village. Children in the community frequently harbour chronic, asymptomatic P. falciparum, *P. vivax*, *P. malariae*, and P. ovale [28]*.* Analysis of the protective effect of α^+^-thalassaemia against severe manifestations of malaria accounted for the matched pair design. Children attending clinics with P. falciparum parasitaemia ≥10,000/μl and no clinical or laboratory features of severe malaria or an alternative cause of an acute febrile illness were also recruited. To investigate the effect of the haematological characteristics according to α^+^-globin genotype on anaemia associated with malaria infection of varying severity, we have pooled children from our original clinic and hospital malaria groups together.

Venous blood was collected from all children at presentation. Thick and thin blood films were prepared, stained with Giemsa, and examined by microscopy for the presence of malaria parasites. Blood collected into EDTA was used for measurement of Hb, erythrocyte count, MCV, and MCH (Coulter MD8 instrument, Coulter Electronics). These measurements were done promptly after sample collection, and reliability of results was ensured by participation in the Coulter instruments quality control scheme. P. falciparum density per microlitre of whole blood was calculated accurately by using the individual measured leukocyte count and parasites per 200 leukocytes counted in thick films. These calculations of parasitaemia differ from standard malariological methods that use only a population average leukocyte count to calculate parasites per microlitre of blood. Percentage parasitaemia was calculated by dividing an individual child's P. falciparum count per microlitre by the number of erythrocytes per microlitre of whole blood measured for that child.

Informed consent was obtained from all individuals and/or their parents or guardian. The study was approved by the Medical Research Advisory Committee of PNG, the Central Oxford Research Ethics Committee, and the Oxford Tropical Research Ethics Committee.

### Data Analysis

Analysis of all haematological data revealed a linear relationship between Hb and erythrocyte count (Figure S1). Children with acute malaria, regardless of conventional severity definitions, demonstrated a reduction in erythrocyte counts and Hb concentrations compared to community control children (Figure S1). Given that Hb concentration was used as a definition for severe disease, and the continuous nature of Hb data, we decided to pool acute malaria data for the purpose of analysis. The association of malaria severity and α^+^-thalassaemia genotype with categorical variables were assessed using chi-squared tests or Fisher's exact test, and continuous data by Mann Whitney U or Kruskal-Wallis tests. The association of α^+^-thalassaemia genotype with risk of SMA was assessed using logistic regression. SPSS (for Windows Release 13.0. 2004, SPSS) was used for data analysis.

### Mathematical Model

Children who are homozygous for α^+^-thalassaemia have smaller erythrocytes containing less Hb and a greater erythrocyte count than children of normal genotype both when living in the community and during episodes of acute malaria. We proposed that this haematological profile protects against SMA because homozygous children lose less Hb from their total erythrocyte pool of Hb, for a given degree of erythrocyte loss, compared with those of normal genotype. A simple model was developed, using data from community children, which predicts total Hb concentration after acute malaria infection as a function of three parameters; (1) baseline level of Hb, (2) MCH, and (3) the reduction in erythrocyte count during a malaria infection. The linear equation *y*_i_ = *b* − *m*_i_ *x* was used to predict Hb concentrations where *y*_i_ refers to predicted Hb concentration in the *i*th child, *b* is the observed genotype-specific median Hb concentration observed in community children prior to acute malarial infection, *m*_i_ represents the observed genotype-specific MCH in the *i*th child, and *x* represents a fixed number of erythrocyte loss during a malaria infection. Values for *b* and *m*_i_ were taken from values observed in the community children, among which all α^+^-thalassaemia genotypes were represented. A range of values for *x* were imputed into the equation in order to derive a slope line for total Hb by reduction in erythrocyte count; separate slope lines were developed for each α^+^-thalassaemia genotype.

## Results

### Characteristics of Study Population

We reanalysed the data from 547 children with acute malaria (median [interquartile range] age 3.0 years [1.8–4.7]; 53.7% male) and 280 children living in the community (3.1 years [1.7–4.5], *p* = 0.81; 53.3% male, *p* = 0.88) who had participated in the earlier study [15].

### Haematological Indices by Disease Status and α^+^-Thalassaemia Genotype

The frequency of α^+^-thalassaemia was high (>80%) in this population (Table 1). Total erythrocyte count was found in the order of normal < heterozygote < homozygote within each clinical group (*p* ≤ 0.001) (Table 1). Values for MCH and MCV were found in the opposite order, i.e., normal > heterozygote > homozygote (*p* < 0.001) (Table 1). As noted in previous studies in Melanesia [2,3], erythrocyte counts, MCH, and MCV were age-dependent (unpublished data). Age was distributed equally among α^+^-thalassaemia genotypes in community children (*p* = 0.17) and those with acute malaria (*p* = 0.98), so it was not a confounder in the analysis presented here. Furthermore, adjustments for age made no difference in the association between α^+^-thalassaemia genotype and haematological indices.

**Table 1:**
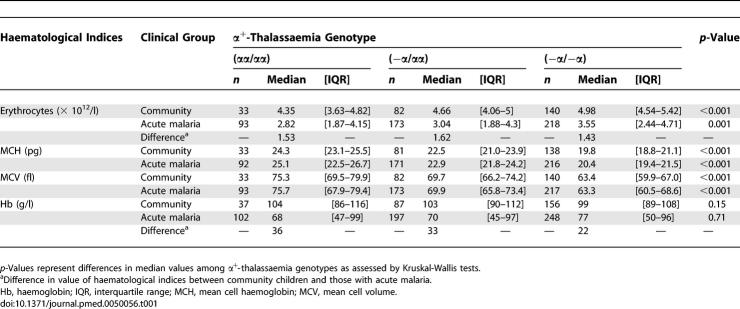
Haematological Indices in Community Children and Children with Acute Malaria According to α^+^-Thalassaemia Genotype

Although Hb concentration was found in the order of normal > heterozygote > homozygote in children living in the community, the increased erythrocyte count in children with α^+^-thalassaemia appeared to compensate somewhat for the microcytosis as there was no significant difference in Hb concentrations according to genotype (*p* = 0.15) (Table 1). Interestingly, the order in Hb concentration was reversed in children with acute malaria, i.e., normal < heterozygote < homozygote (Table 1). The difference in median Hb concentration between community controls and acute malaria groups was lower in children homozygous for α^+^-thalassaemia than in children of normal genotype (Table 1).

**Figure 1:**
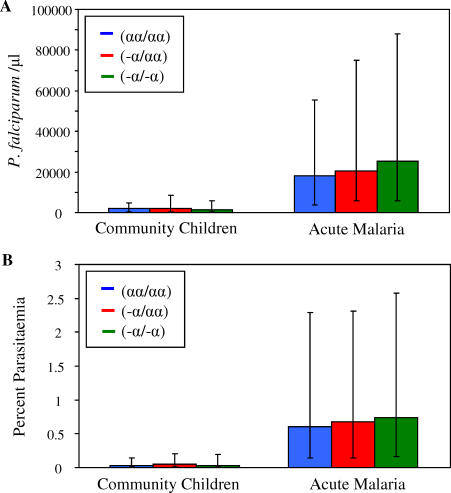
Parasite Density in Community Children and Those with Acute Malaria According to α^+^-Thalassaemia Genotype Parasite density is represented by (A) the number of P. falciparum-infected erythrocytes per microlitre of blood and (B) the proportion of P. falciparum-infected erythrocytes. Values are median (interquartile range). There was no statistically significant difference in parasite density or percent parasitaemia among α^+^-thalassaemia genotypes, in either children living in the community or those with acute malaria (*p* ≥ 0.3).

This difference in Hb loss according to genotype could not be accounted for by differences in parasitaemia. Children with acute malaria had significantly higher P. falciparum densities (18,880 parasites/μl [3,142–73,950]) than children living in the community (*n* = 104, 1,974 parasites/μl [308–9,233], *p* < 0.001). Parasite counts per microlitre of blood and percent parasitaemia (which adjusts for genotype-specific differences in erythrocyte count) were similar among α^+^-thalassaemia genotypes in both children living in the community and those with acute malaria (Figure 1, *p* ≥ 0.3). The median erythrocyte count was 1.5 × 10^12^/l lower in children with acute malaria (3.27 × 10^12^/l [2.11–4.44]) than community children (4.78 × 10^12^/l [4.24–5.24], *p* < 0.001) and, in contrast to Hb concentration, this difference in erythrocyte count was remarkably similar across all genotypes (Table 1).

### Decrease in Hb According to Genotype-specific Differences in MCH

**Figure 2:**
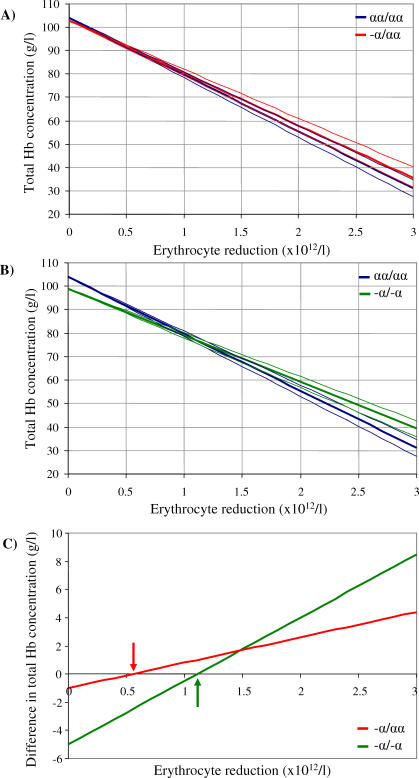
Consequence of Reduction in Erythrocyte Count on Total Haemoglobin Concentration According to α^+^-Thalassaemia Genotype The linear relationship between Hb concentrations and reduction in erythrocyte count can be described by the following linear equation: *y*_i_ *= b − m*_i_ *x*, where *y*_i_ refers to predicted Hb concentration in the *i*th child, *b* is the observed Hb value in the community children prior to acute malaria infection taken from Table 1, *m*_i_ represents observed MCH in the *i*th child, and *x* represents the reduction in erythrocyte count. (A and B) Predicted total Hb concentration of children of normal genotype together with (A) α^+^-thalassaemia heterozygotes and (B) α^+^-thalassaemia homozygotes during reductions in erythrocyte count. Thick lines represent median values and thin lines represent the interquartile range. The equations are *y =* 104 − 24.3*x* [*y =*104 − 25.5, *y =* 104 − 23.1*x*] for normal individuals; *y =* 103 − 22.5*x* [*y =* 103 − 23.9, *y =*103 − 21.0*x*] for heterozygous; and *y =* 99 − 19.8*x* [*y =* 99 − 18.8*x*, *y =* 99 − 21.1*x*] for children homozygous for α^+^-thalassaemia. (C) Difference in total predicted Hb (*y*) between those of normal genotype and heterozygous children (*y =* 1.8*x* − 1, red line, data from [A]), and between those of normal genotype and homozygous children (*y =* 4.5*x* − 5, green line, data from [B]). The crossover point where heterozygous individuals have a greater Hb concentration relative to those of normal genotype is an erythrocyte reduction of 0.56 x10^12^/l (red arrow). The crossover point where homozygous individuals have a greater Hb concentration relative to those of normal genotype is an erythrocyte reduction of 1.1 x10^12^/l (green arrow). These crossovers are also seen in (A) and (B) but are better visualised here.

MCH was 20% lower in community children homozygous for α^+^-thalassaemia than in individuals of normal genotype (*p* < 0.001, Table 1). Thus, for a given degree of reduction in erythrocyte count, children who are homozygous for α^+^-thalassaemia will release 20% less Hb from the erythrocyte pool of Hb than those of normal genotype. Using the MCH values for each child living in the community and on the basis of the linear relationship between Hb and erythrocyte count represented by the linear equation *y*_i_ = *b* − *m*_i_ *x*, we calculated the reduction in Hb concentration for the range of differences in erythrocyte count observed between community controls and acute malaria (Figure 2A and 2B). This simple model predicted that at degrees of reduction in erythrocyte count <1.1 × 10^12^/l, α^+^-thalassaemia homozygotes had a lower Hb concentration compared to normal individuals. In contrast, at levels of reduction in erythrocyte count >1.1 × 10^12^/l, children homozygous for α^+^-thalassaemia had a higher Hb concentration than those of normal genotype (Figure 2B and 2C).

### Erythrocyte Count Cutoff for SMA According to Genotype

**Figure 3:**
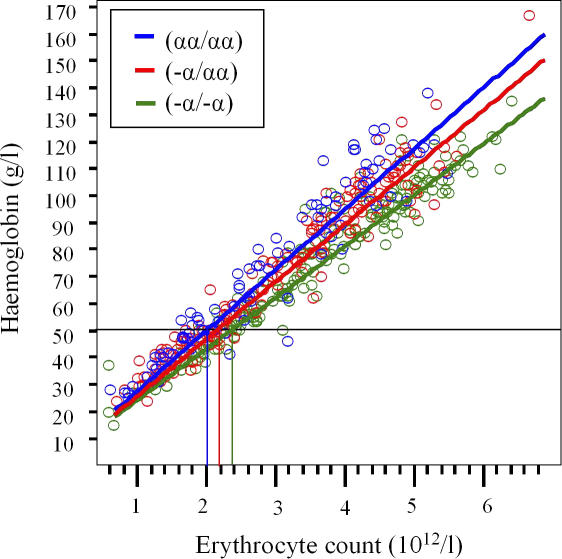
Genotype-Specific Erythrocyte Cutoffs for Severe Malarial Anaemia Data points represent haemoglobin concentration and erythrocyte count values for all children with acute malaria. Genotype-specific lines of best fit have been generated for the association of haemoglobin with erythrocyte count. The horizontal black line represents the cutoff for severe malarial anaemia (haemoglobin = 50 g/l). The coloured vertical lines represent the genotype-specific erythrocyte cutoffs for severe malarial anaemia based on a haemoglobin concentration of 50 g/l.

Children homozygous for α^+^-thalassaemia with lower MCH and increased erythrocyte count may require a greater reduction in erythrocyte count to develop SMA, defined as Hb concentration <50 g/l [26]. To determine the genotype-specific erythrocyte count cutoff for a Hb concentration of 50 g/l, erythrocyte count was plotted against Hb concentration for all children with acute malaria and a line of best fit drawn for each genotype (Figure 3). The erythrocyte count cutoff for SMA was determined to be 2.4 × 10^12^/l in homozygous α^+^-thalassaemia, 2.2 × 10^12^/l in heterozygotes, and 2.0 × 10^12^/l in normal individuals (Figure 3), because of differences in MCH values among genotypes.

For each child living in the community, the reduction in erythrocyte count required to reach an Hb concentration of 50 g/l was calculated. The median (interquartile range) erythrocyte loss was higher in homozygous α^+^-thalassaemia (2.58 × 10^12^/l [2.14–3.02]), than heterozygous α^+^-thalassaemia (2.46 × 10^12^/l [1.86–2.8], *p* = 0.08), and normal individuals (2.35 × 10^12^/l [1.63–2.82], *p* = 0.02). In other words, children homozygous for α^+^-thalassaemia would require 10% greater reduction in erythrocyte count than children of normal genotype, for Hb concentration to fall to 50 g/l. This result should be considered in relation to our observation that similar degrees of reduction in erythrocyte count were found among α^+^-thalassaemia genotypes in acute disease, relative to community controls (∼1.5 × 10^12^/l; Table 1).

To determine whether microcytosis and increased erythrocyte count could explain the protection observed against SMA in α^+^-thalassaemia homozygotes, we applied the reduction of 2.35 × 10^12^/l erythrocytes (the median reduction in erythrocyte count observed in children of normal genotype in the estimated transition between community control and SMA) to all children living in the community. The proportion of community children predicted to develop SMA, defined by the genotype-specific erythrocyte count definitions for SMA, was lower in children who were homozygous for α^+^-thalassaemia (*n* = 50, 35.7%) than in heterozygous children (*n* = 36, 43.9%), and those of normal genotype (*n* = 17, 51.5%). Compared with normal children, the risk of a community child being defined as having SMA was 0.52 (95% confidence interval [CI] 0.24–1.12, *p* = 0.09) in α^+^-thalassaemia homozygotes and 0.74 (95% confidence interval [CI] 0.33–1.66, *p* = 0.46) in heterozygotes for this degree of reduction in erythrocyte count. This simple model based on observed haematological data predicted that children who were homozygous for α^+^-thalassaemia would be 48% less likely to develop SMA than children of normal genotype.

## Discussion

The reasons for the microcytosis and relatively high erythrocyte count in carriers for alpha or beta thalassaemia are not understood. It has been proposed that it reflects an increased number of terminal cell divisions during erythropoiesis, due to the combination of defective haemoglobinisation of the erythrocytes and a highly proliferative bone marrow [29]. We investigated whether the microcytosis and increased erythrocyte count associated with α^+^-thalassaemia may be a haematological advantage in the face of the estimated >30% reduction in erythrocyte count, which we observed in PNG children with acute malaria.

Modelling observed data, we show that a lower concentration of Hb per erythrocyte and a larger population of erythrocytes may be a biologically advantageous host strategy against a pathogen that significantly lowers erythrocyte count. We define a crossover point of reduction in erythrocyte count due to acute malaria (1.1 × 10^12^/l) at which microcytosis and an increased erythrocyte count becomes an advantage to the host. This erythrocyte cutoff is considerably lower than the median erythrocyte reduction we estimated to be associated with acute malaria (∼1.5 × 10^12^/l). This result would account for the reversal of Hb concentrations in children with acute malaria (normal < heterozygote < homozygote), a phenomenon that has been noted previously [16,29]. We show that the erythrocyte count associated with this Hb concentration cutoff is genotype-specific and found in the order normal < heterozygous < homozygous. We also show that children homozygous for α^+^-thalassaemia would require a greater reduction in erythrocyte count to reach the SMA cutoff. We therefore propose that a higher microcytic erythrocyte count in children homozygous for α^+^-thalassaemia enables them to maintain their Hb concentration above the 50 g/l threshold, thereby reducing the risk of SMA. Indeed, given the degree of malaria haemolysis associated with SMA in normal individuals, children who were homozygous for α^+^-thalassaemia would be 48% less likely to develop SMA than children of normal genotype. This result suggests that microcytosis and increased erythrocyte count contribute considerably to the 66% protection against SMA observed in individuals homozygous for α^+^-thalassaemia in this population [18].

We found no significant difference in parasite counts per microlitre of blood or percent parasitaemia among α^+^-thalassaemia genotypes in community children, nor in those with acute malaria, in concordance with earlier studies [15–19]. This finding is surprising given that the increased erythrocyte count in homozygous children may be expected to lower the proportion of infected erythrocytes. After adjustments for individual erythrocyte counts, the variance of parasitology data was more comparable among α^+^-thalassaemia genotypes compared to parasites per microlitre of blood. It is possible that density-dependent mechanisms [28] may regulate parasitaemia and reduce genotype-specific differences.

Malarial anaemia is attributed to parasite-induced haemolysis, destruction of unparasitised erythrocytes, and dyserythropoiesis [30]. It has been proposed that individuals with α^+^-thalassaemia have increased phagocytosis of erythrocytes [31–33] and expanded erythroid marrow [34], but the balance between erythrocyte survival and production among α^+^-thalassaemia genotypes is unknown. The model shown in Figure 2 predicts the total Hb concentration for a given reduction in erythrocyte count and fixed MCH. It does not take into account potential differences in the balance between destruction of erythrocytes and production of reticulocytes among α^+^-thalassaemia genotypes, and this area merits further research.

Other mechanisms may also contribute to the protection against SMA. α^+^-Thalassaemia homozygosity has also been shown to be associated with low complement receptor 1 (CR1) expression [35]. This molecule has been shown to be important in the binding of infected erythrocytes to noninfected erythrocytes (rosetting) [36], as well as the clearance of erythrocytes as they age [37]. Increased rosetting has been implicated in the pathogenesis of severe disease [38]. While α^+^-thalassaemic erythrocytes are less likely to form rosettes in vitro [39,40], further studies are required to investigate the contribution of complement receptor 1 (CR1) to the protection α^+^-thalassaemia affords against SMA. Both polymorphisms could synergise to minimise the reduction in erythrocyte count during acute disease.

Malaria is a complex multisystem disorder, and in children, in addition to anaemia, important severe manifestations include cerebral malaria, acidosis, and hyperlactataemia [41]. No protection of α^+^-thalassaemia was observed against malaria coma in the matched-pair analysis in the PNG study [18], whereas studies in Africa have shown a protective effect against cerebral malaria of heterozygosity [19,20,23] and homozygosity [20]. There is also evidence that children heterozygous and homozygous for α^+^-thalassaemia are protected against acidosis [18,20,23] and hyperlactataemia [18]. Comparison of the effects of potential protective factors between studies is complicated because the criteria used to define severe manifestations of malaria often differ between studies. Also, the pathophysiological mechanisms underlying severe malaria may differ between populations. It is unclear how anaemia may be related with these other clinical pathologies but a key role for pro-inflammatory cytokines has been implicated in all clinical sub groups [41]. Hb is an extremely toxic molecule. Outside the erythrocyte and its constituent antioxidant defence systems, the oxidative potential of Hb can cause substantial oxidative tissue damage and release of pro-inflammatory cytokines such as tumour necrosis factor-α [42,43]. Children homozygous for α^+^-thalassaemia release less Hb per erythrocyte during haemolysis and therefore it is plausible that they do not stimulate pro-inflammatory responses as readily as do those of normal genotype. Interestingly, children homozygous for α^+^-thalassaemia have also been shown to be protected against severe nonmalaria disease in this population [18]. Lower Hb concentrations per cell may reduce inflammation during any disease-related haemolysis.

We propose that the microcytosis and higher erythrocyte count associated with α^+^-thalassaemia homozygosity is a selective advantage against SMA. In contrast, the parasite/erythrocyte interaction hypothesis cannot account for protection against SMA as it implies differences in parasite counts by genotype, which we failed to find in any disease state. Whilst our analysis has focused on P. falciparum, this haematological mechanism may protect against other *Plasmodium* species, such as P. vivax. P. vivax infection can be associated with severe anaemia, and it is interesting to note that the highest frequencies of α^+^-thalassaemia are found in areas where P. vivax is prevalent [1,44]. Since other common disorders of Hb that appear to provide relative protection against severe malaria, notably carriers of beta thalassaemia and homozygous Hb E, also have relatively high microerythrocyte counts [1,45], this haematological mechanism of protection may have broader implications for our understanding of the selection of these host erythrocyte polymorphisms by malaria.

## Supporting Information

Figure S1 The Association of Hb Concentration and Erythrocyte Count in Study Children Hb is positively associated with erythrocyte count, *r* = 0.94, *p* < 0.001. Horizontal and vertical lines represent observed median Hb (g/l) and erythrocyte count respectively for each definition of malaria. Solid green line represents acute malaria, dashed green line all hospital malaria. Median (interquartile range) Hb and erythrocyte counts are as follows: severe malaria, 47 g/l (38–79), 2.12 × 10^12^/l (1.58–3.52); all hospital malaria, 61 g/l (44–91), 2.76 × 10^12^/l (1.89–4.09); acute malaria, 72 g/l [47–97], 3.27 × 10^12^/l [2.11–4.44]; mild malaria, 90 g/l [67–103], 4.03 × 10^12^/l [3.12–4.84]; community children, 100 g/l [88–109], 4.78 × 10^12^/l [4.24–5.24].
(158 KB JPG)

## Abbreviations

Hb: haemoglobin
MCH: mean cell haemoglobin
MCV: mean cell volume
PNG: Papua New Guinea
SMA: severe malarial anaemia
